# The Role of Viruses in the Glioma Tumor Microenvironment: Immunosuppressors or Primers for Anti-Tumor Immunity?

**DOI:** 10.3390/cancers17121984

**Published:** 2025-06-14

**Authors:** Anna J. Hudson, Jay Chandar, Muhammet Enes Gurses, Thomas Malek, Ashish H. Shah

**Affiliations:** 1 Section of Virology and Immunotherapy, Sylvester Comprehensive Cancer Center, Miller School of Medicine, University of Miami, Miami, FL 33136, USA; annahudson@med.miami.edu (A.J.H.); jchan119@med.fiu.edu (J.C.); 2 Department of Microbiology and Immunology, Miller School of Medicine, University of Miami, Miami, FL 33136, USA; tmalek@med.miami.edu; 3 Department of Neurosurgery, Miller School of Medicine, University of Miami, Miami, FL 33136, USA; megursesmd@gmail.com

**Keywords:** glioblastoma, viruses, tumor microenvironment, CMV, immunotherapy, herpesviruses

## Abstract

The WHO estimates that nearly 10–15% of cancers have a known viral etiology, although this number is likely an underestimate. In glioblastoma (GBM), the most common primary brain malignancy, viral associations have been proposed and investigated without a definitive etiology. Viral–host interactions are known to alter cellular growth and stem cell programming, as well as modulate innate immune signaling. However, in GBM, the multifaceted role of endogenous or exogenous viral expression remains unclear. Here, we provide a review of common viral associations in GBM and discuss how these viruses modulate intrinsic cellular processes to enhance anti-viral immune response or suppress anti-tumor immunity.

## Simple Summary

Viral infections have been linked to the development of several cancers, including glioblastoma—the most common primary brain tumor. This review summarizes current research on how different viruses may influence glioblastoma progression and patient outcomes. We also explore therapeutic strategies that target these viruses as potential strategies to improve treatment for this aggressive disease.

## 1. Introduction

Thirteen percent of all new cancer diagnoses are estimated to be linked to infections [1]. Of these infections, several viruses have been specifically associated with the development of brain tumors [1,2]. Most research into the viral etiology of gliomas has focused on CMV. Nevertheless, several other viruses known for their oncogenic or immunosuppressive properties may influence GBM (grade IV glioma) incidence, progression, and overall survival (OS). The prognosis of GBM remains poor with a median survival of fourteen months from the time of diagnosis and a 5-year survival rate of less than 5%, even with the current standard of care, which includes surgical resection when possible and chemoradiation [1,2,3,4]. Thus, the need to develop alternative treatment options for these patients remains imperative. Immunotherapies such as immune checkpoint inhibitors and CAR T cells have shown promise in improving outcomes for other tumor types. However, immunotherapies and other emerging treatments have faced challenges in GBM [5,6]. A limitation is GBM’s highly immunosuppressive tumor microenvironment (TME). The TME of GBM is characterized by highly exhausted infiltrating CD8^+^ T cells, low MHC class I expression, and large numbers of regulatory T cells (Tregs) and myeloid-derived suppressor cells (MDSCs) [5,6,7]. Several solid tumors with an immunosuppressive TME are also associated with oncogenic viral infections [1]. Therapies that target these viruses within the GBM TME may simultaneously function to decrease the malignant potential of tumor cells, while increasing the anti-tumor immune response. Here we review the role of tumor-associated HTLV-1, HIV, HSV-1, VZV, EBV, CMV, HHV-6 and other viruses in the local and systemic immune responses as they relate to oncomodulation of gliomas. We examine these viruses due to their widespread prevalence and/or their known malignant potential.

## 2. HTLV-1

A total of 15–20 million people worldwide are infected with the retrovirus Human T-Lymphotropic Virus 1 (HTLV-1), which is a main cause of adult T cell leukemia (ATL), an aggressive hematologic malignancy of CD4^+^ T cells [8,9]. While the precise mechanisms of HTLV-1-induced ATL tumorigenesis remain unclear, oncogenic viral genes *tax* and *HBZ* alter host oncogenic pathways, including NF-κB, CREB, and AKT [9]. Outside of ATL, HTLV-1 is also associated with endometrial adenocarcinoma, hepatocellular carcinoma, non-small-cell lung cancer, and small-cell lung cancer [10].

In addition to its role in malignancies, HTLV-1 infection also plays a role in neuropathogenesis. HTLV-1 causes the chronic progressive myelopathy tropical spastic paraparesis/HTLV-1-associated myelopathy (TSP/HAM), which is an inflammatory demyelinating disease characterized by lymphocytic infiltration of the central nervous system (CNS) [11]. The blood–brain barrier is disrupted in HAM/TSP patients, and the brains of some HAM/TSP patients have HTLV-1-infected astrocytes (detected by viral mRNA) [8,12,13,14]. Further, HTLV-1 infected T cells have an increased ability to cross the blood–brain barrier into the CNS, where they then bind to astrocytes [15]. The neuropathology that occurs in HAM/TSP is thought to result from the interactions between cells of the CNS and infiltrating immune cells. Crosstalk between HTLV-1-infected T cells and astrocytes alters astrocytic function by decreasing extracellular glutamate uptake, decreasing expression of glial transporters GLAST and GLT-1, and increasing production of inflammatory cytokines [11].

Given HTLV’s oncogenic nature and its ability to infect astrocytes, a few studies have explored a potential role of HTLV-1 in gliomagenesis. In vitro, HTLV-1-infected glioma stem cells (GSCs) alter glutamate metabolism and increase production of granulocyte-macrophage colony-stimulating factor (GM-CSF), Tumor Necrosis Factor-alpha (TNF-α), IL-1α, and IL-6 [14,16]. GM-CSF contributes to an immunosuppressive GBM TME, and TNF-α and IL-1 promote tumor invasion, tumor cell migration, and angiogenesis [17,18,19]. IL-6 is a STAT3-activating cytokine that further contributes to angiogenesis and tumor cell proliferation, as well as radiation resistance in GBM [20,21]. Further, KEGG pathway analysis shows that HTLV-1 infection and Tax protein expression are common factors in the development of different non-hematologic malignancies, including GBM [10]. Additionally, upregulation of CDKN1A (Cyclin-dependent kinase inhibitor 1A) is associated with HTLV-1 infection and chemoresistance in GBM [22]. Thus, despite the limited information about HTLV-1 in gliomas, initial studies support further investigation to better understand the role of this virus in the GBM TME.

## 3. HIV

Human immunodeficiency virus (HIV) infection affects approximately 40 million people worldwide and results in several acute and chronic immune changes that are associated with the development of acquired immunodeficiency syndrome (AIDS) [23]. HIV has multiple secondary effects in the CNS and is associated with significant differences in outcomes for people living with HIV (PLWH) diagnosed with GBM [7]. Additionally, HIV infection is highly correlated with the development of primary brain tumors, most notably primary CNS lymphoma [24]. This pathogenesis is related in part to the immunosuppressed state of PLWH and co-infections with other oncogenic viruses [24]. Though the role of HIV in the development of primary gliomas is not clear, certain studies demonstrate a potential for increased incidence of GBM after HIV infection [25]. For instance, GBM occurs at a younger mean age (39 years) in infected individuals than in the general population (61 years) and at a 5.4–45-fold higher frequency [7,24]. Data from our lab is also consistent with the notion that PLWH diagnosed with GBM have significantly worse OS [26]. Further investigation of the oncologic and immunologic mechanisms associated with HIV will allow us to contextualize its impact on the local immune milieu in glioma and provide insight into its impact on GBM patient outcomes.

HIV characteristically infects CD4^+^ T cells, but it also infects dendritic cells (DCs), macrophages, B cells, natural killer (NK) cells, immature bone marrow and thymic precursors, as well as astrocytes and microglia. Subsequent transcription of the HIV RNA genome by reverse transcriptase (RT) and integration into the host genome by integrase establishes a persistent infection [27]. Systemic HIV infection activates toll-like receptors and cells of the innate immune system (DC, monocytes, NK cells) which create widespread cytokine secretion. Stimulation of the immune system, in turn, promotes HIV replication in infected immune cells [28]. However, the adaptive immune response is incapable of curing HIV infection, largely due to HIV-mediated depletion of CD4^+^ T cells. This leaves the host in an immunosuppressed state leading to greater risk of co-infections, viral-induced oncogenesis, and neuropathogenesis [29].

**Figure 1:**
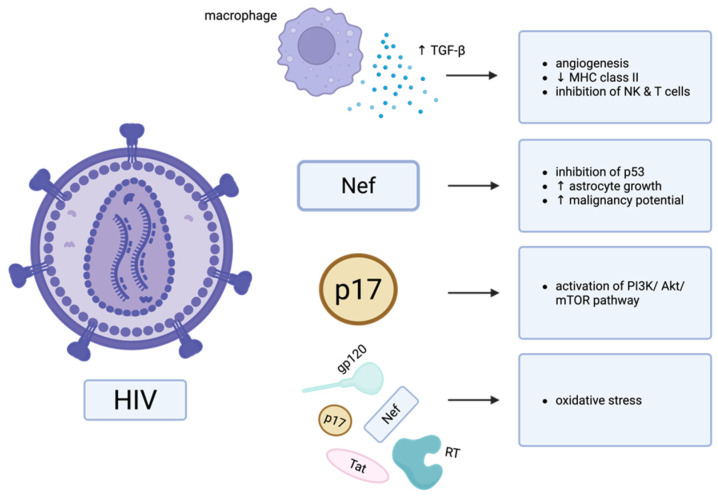
Systemic infection with HIV leads to local changes in the brain that may promote the formation of brain tumors, leading to increased incidence of GBM in PLWH compared to the general population. The release of immunosuppressive cytokines from infected immune cells, oxidative stress induced by HIV proteins, and activation of oncogenic pathways by HIV proteins may contribute to these effects. Created in BioRender. Hudson, AJ. (2025) https://BioRender.com/9uqqeoa (accessed on 12 June 2025).

Locally, HIV infection alters cellular function and cytokine production (Figure 1). HIV infection of macrophages in the brain leads to overexpression of TGF-β2, an immunosuppressive cytokine in PLWH. This cytokine is highly expressed in GBM, and is not present in the normal brain [7]. TGF-β2 promotes angiogenesis, downregulates MHC class II expression, and inhibits NK and T cell effector function [7]. Further, many HIV-derived proteins play a role in PLWH’s significantly increased risk for developing malignancies. For instance, envelope protein gp120, accessory protein negative factor (Nef), matrix protein p17, transactivator of transcription (Tat), and reverse transcriptase (RT), are released from infected cells into the local environment and induce oxidative stress, which in turn induces malignant transformation of surrounding cells. Oxidative stress is also associated with increased number of GSCs within the GBM TME [24,30,31]. These GSCs further contribute to proliferation and tumorigenesis in GBM, as well as immunosuppression in the TME [32,33]. Further, Nef inhibits the apoptotic function of tumor suppressor p53 and alters astrocyte phenotype in culture, promoting cell growth and a morphology resembling neoplastic cells [7,34]. In vivo, Nef stimulates tumor formation in neural stem cells and increases the malignancy potential of lower grade astrocytomas [7]. HIV’s p17 activates the PI3K/Akt/mTOR pathway, which is critical for the development and malignant progression of several cancers [35]. Additionally, HIV-mediated immunosuppression may lead to viral-based oncogenesis due to co-infection with other viruses, including hepatitis B virus (HBV), human papillomavirus (HPV), Kaposi’s sarcoma-associated herpes virus (KSHV), and Epstein–Barr virus (EBV) [24,26]. For example, in coinfection with EBV, HIV’s p17 binds CXCR2, allowing EBV’s oncogenic latent membrane protein 1 (LMP1) to enter cells to promote cell proliferation [36]. Collectively, these effects together promote an oncogenic microenvironment and may impact the development of primary gliomas in patients with HIV.

HIV-targeting antiretroviral therapies (ART) are under investigation in preclinical studies and clinical trials of GBM patients without HIV as potential repurposed drugs. Since HIV protease inhibitors decrease the expression of matrix metalloproteases (MMPs) and impair tumor cell migration and invasion, a 2010 phase II clinical trial explored combining ritonavir and lopinavir on GBM patient outcomes [37]. However, the study did not meet its efficacy endpoint as out of nineteen patients enrolled, only one patient had complete tumor regression, and three others exhibited stable disease [37]. Another clinical trial investigated the role of the HIV protease inhibitor nelfinavir in combination with the current standards of care, temozolomide (TMZ) and radiotherapy, based on data that nelfinavir prevents the growth of cancer cell lines by inhibiting the PI3K/Akt/mTOR pathway [38,39]. Though the phase I study showed the safety of the treatments, the median OS of patients was 13.7 months, about the same as the median OS with the current standard treatments [38].

More recent preclinical studies show that other ART drugs inhibit GBM progression. Our lab found that treatment with several different HIV reverse transcriptase inhibitors and protease inhibitors causes decreased GBM cell viability, stemness, and proliferation, with abacavir and lamivudine having especially potent effects [40]. As ART may benefit GBM patients without HIV and evidence that HIV infection exacerbates GBM patients’ prognoses, it remains important to study how ART modulates the immune system in both GBM and HIV disease settings. While the exact relationship between HIV and GBM remains unclear, these studies point to a role for antiretroviral therapy (ART) in the management of GBM in PLWH.

## 4. Herpesviruses

Human herpesviruses (HHVs) are enveloped, double-stranded DNA (dsDNA) viruses, with more than 90% of adults infected with one or more HHV [1,41,42]. HHVs can cause latent infections in hosts, reactivating periodically after primary infection, and most herpesviruses are neurotropic [1,41]. These characteristics make HHVs such as HSV-1, VZV, EBV, CMV, and HHV-6 interesting candidates in studying the viral etiology of GBM.

### 4.1. HSV-1

Herpes simplex virus (HSV-1 or HHV-1) infection is important to consider in patients with glioblastoma because it is highly prevalent. In the United States, an estimated 47.8% of people between 14 and 49 years old are HSV-1 seropositive, and worldwide, HSV-1 seropositivity is reported to be as high as 90% [43,44,45]. Studies investigating the effects of pre-existing systemic HSV-1 infection on the prognosis of GBM patients have yielded mixed results. Evaluation of immunoglobulin G (IgG) levels to the herpesviruses VZV, CMV, EBV, HSV-1, and HSV-2 in 1378 adults diagnosed with glioblastoma found that HSV-1 seropositivity was not associated with altered survival [46]. Conversely, our lab showed a significant reduction in OS in patients with concomitant GBM and herpes simplex encephalitis (HSE), a major complication of HSV-1 infection, compared to a TCGA cohort (8 months vs. 14.2 months) [20,30,47]. Outside of the HSE setting, HSV-1 genetic material has also been found in the brains of seemingly healthy individuals [48,49].

**Figure 2:**
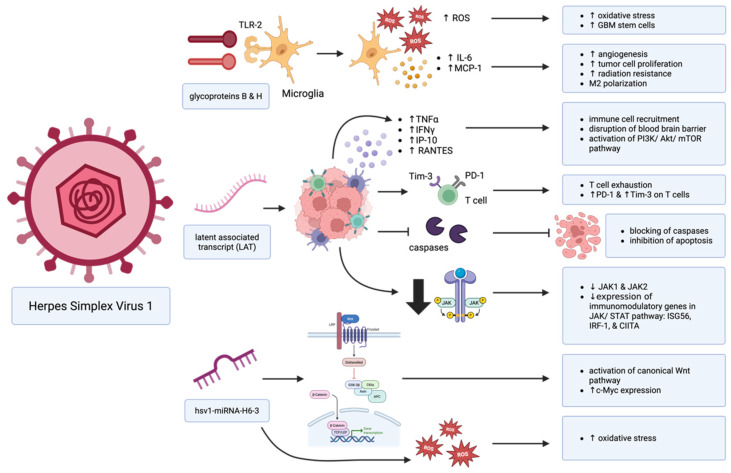
Though not directly oncogenic in nature, HSV-1 infection primes the local brain tissue for the formation of aggressive GBM tumors by inducing oxidative stress, altering the immune landscape to be more suppressive, and indirectly activating oncogenic pathways like Wnt. Created in BioRender. Hudson, AJ. (2025) https://BioRender.com/cnz8uyf (accessed on 12 June 2025).

Though HSV-1 is not directly oncogenic in nature, in immunosuppressed settings like GBM, it has the potential to contribute to tumorigenesis via a few mechanisms (Figure 2). For instance, HSV-1 glycoproteins B and H (gB and gH) bind toll-like receptor 2 (TLR-2) on microglial cells, resulting in the production of reactive oxygen species (ROS) and the upregulation of cytokines such as monocyte chemoattractant protein-1 (MCP-1) and interleukin-6 (IL-6) [50,51]. Excess ROS production can cause free radicals to bind to bases including pyrimidines, purines, and chromatin-associated proteins, resulting in genomic instability, chromosomal modifications, and tumorigenesis [20,52]. Further, MCP-1 increases GBM’s recruitment of microglia and polarizes these immune cells toward their immunosuppressive M2 phenotype [20,53].

In addition, latent infection of HSV-1 is marked by overexpression of latency-associated transcript (LAT) and a unique immune landscape [54]. Latent infection of the trigeminal ganglia (TG) is linked to increased CD8^+^ T cell infiltration and alterations in cytokine levels, including increased TNF-α, interferon-gamma (IFN-γ), interferon gamma-induced protein 10 (IP-10), and RANTES (regulated upon activation, normal T cell expressed and secreted) [54,55]. TNF-α induces apoptosis, polarizes microglia to their proinflammatory M1 phenotypes, and disrupts the blood–brain barrier among other functions [56]. IP-10 (induced by IFN-γ) and RANTES are chemokines involved in the recruitment of immune cells and may recruit T cells to the site of latent infection [54,57,58]. RANTES activates mTOR and PI3K pathways, resulting in increased tumor cell proliferation, growth, and invasiveness [20,59]. Importantly, the T cells present in latent HSV-1 infection demonstrate exhaustion in a LAT-dependent manner, expressing increased PD-1 and TIM-3 [60]. LAT also inhibits apoptosis by blocking activation of proapoptotic caspase proteins [60]. Further, latent HSV-1 infection is associated with downregulation of JAK1, JAK2, and several immunomodulatory genes downstream in the JAK/STAT pathway, such as ISG56 (interferon stimulated gene 56), IRF-1 (interferon regulatory factor 1), and CIITA (class II major histocompatibility complex transactivator) [60]. ISG56 is a negative regulator of IFN-β, IRF-1 is a tumor suppressor that is downregulated in many cancers, and CIITA modulates MHC-II expression [61,62,63].

Viral microRNAs (miRNAs) released by HSV-1 also appear to play a role in the GBM TME [64]. For instance, hsv1-miR-H6-3p is upregulated in GBM and decreases expression of the genes EPB41L1 (erythrocyte membrane protein band 4.1) and SH3PXD2A (SH3 and PX Domains 2A) [64]. EPB41L1 is critical for cell adhesion and migration, and its downregulation is associated with poor survival in various cancers [65,66]. Further, its downregulation activates the classical Wnt pathway and c-Myc expression [66]. Dysregulated Wnt signaling is associated with various malignancies, as is aberrant expression of Wnt target gene c-Myc [67]. SH3PXD2A regulates superoxide-generating NADPH oxidase activator activity [64]. Thus, its downregulation potentially exacerbates oxidative stress. In sum, latent HSV-1 infection results in oxidative stress, alteration of cytokine levels, chronic inflammation, and T cell dysregulation, which may, in turn, contribute to an increased potential for glial cell transformation.

Several studies have explored the effect of HSV-1 seropositivity on response to GBM treatments, especially oncolytic HSV (oHSV) treatment. Because it activates immune responses, HSV-1 has been heavily studied as a virotherapy for immunologically cold GBM. oHSV therapy promotes tumor cell death, in part, by infecting and replicating in tumor cells, leading to tumor cell lysis [68]. When tumor cells die, they release both tumor-associated antigens and viral antigens, intensifying the anti-tumor immune response [68,69]. oHSV therapy of glioma results in increased recruitment of CD45^+^ immune cells, including CD8^+^ T cells, NK cells, and M1 macrophages, all of which combine to promote tumor cell death [68]. The genetically modified oHSV Delytact was conditionally approved in Japan for treatment of GBM in 2021, which raises the question of how pre-existing HSV-1 infection affects patients’ response to treatment [68,70].

A rat model of GBM showed that pre-existing HSV-1 immunity diminishes oHSV infection of tumor cells [71]. A clinical trial exploring injections of the oHSV CAN-3110 into recurrent GBM tumors showed that positive HSV-1 serology was significantly associated with enhanced immune clearance of CAN-3110 and improved survival in patients [72]. HSV1-seropositive patients with IDH-wild-type GBM had a median OS of 14.2 months, while patients with negative HSV-1 serology had a mOS of 7.8 months [72]. This trend was replicated in a small cohort of patients with IDH mutant tumors (a molecular subtype of GBM that is less aggressive than IDH-wild-type tumors) [72]. Statistical analysis showed that HSV-1 serology is a highly significant independent predictor of survival [72]. This study also found that tumors of HSV-1 seropositive patients had higher T cell receptor beta chain (TCRβ) diversity than tumors of seronegative patients [72]. This finding is important because low TCRβ diversity in tumor-adjacent tissue and lymph nodes is associated with poor clinical prognosis in multiple malignancies [73,74]. Further, a diverse TCRβ repertoire is associated with improved response to programmed-death 1 (PD-1) treatment in melanoma patients [75]. Overall, further investigation of the effects of HSV-1 infection of the CNS and HSV-1 seropositivity on outcomes in glioma and other cancers is warranted given HSV-1’s high prevalence in the world and the expanding use of oHSV virotherapies.

### 4.2. VZV

Varicella zoster virus (VZV), or human herpesvirus 3 (HHV-3), causes chickenpox and is neurotropic, establishing latency in the cranial nerve and dorsal root ganglia [1,76]. VZV reactivation occurs in 10–20% of people and can lead to shingles, or, more rarely, serious neurological conditions like encephalitis and myelitis [1,76]. Due to its neurotropism, VZV is relevant to studies of viral infection and gliomagenesis. Notably, VZV is the only herpesvirus consistently associated with a reduced risk of glioma [76]. Across studies, both self-reported chickenpox history and VZV-specific IgG levels are lower in GBM patients versus control patients [77,78,79,80]. Additionally, a study of 1378 GBM patients found that VZV seropositivity was associated with improved survival [46]. Together, these findings suggest that prior VZV infection may confer protection against GBM development and progression. While the underlying mechanism of protection remains unclear, one theory is that VZV antibodies cross-react with glioma antigens or other neurotropic oncogenic viruses [76]. Like HSV-1, VZV has been proposed as a novel virotherapy candidate for GBM given its neurotropism, rapid division, and lysis of malignant glioma cells in vitro; thus, elucidating VZV’s antitumorigenic role in the GBM TME could help guide its future therapeutic use [76].

### 4.3. EBV

Epstein–Barr virus (EBV/HHV-4) was the first recognized human oncovirus and commonly results in mononucleosis characterized by pharyngitis, fever, and fatigue [3]. Reactivation of the latent stage of the virus is strongly associated with the development of B-cell lymphomas, nasopharyngeal carcinomas, and gastric carcinomas [3,81,82,83]. This oncogenic function can be partially explained by the ability of EBV to alter several cellular survival pathways, such as NF-κB, MAPK, PI3KA, JAK/STAT, and Notch [84].

While the role of EBV in the above cancers has been well-established, its role in gliomagenesis is just starting to be explored. EBV infection in the CNS often occurs in immunocompromised patients and is associated with a number of CNS pathologies, such as acute encephalitis, cerebellar ataxia, demyelinating disease, and meningitis [3]. Additionally, EBV is a potential cause of primary CNS lymphomas [3]. While a mechanistic role of EBV infection in gliomas has not been clearly defined, many studies have investigated the prevalence and prognosis of EBV in gliomas [85]. One study explored the OS of EBV^+^ patients with GBM versus EBV^−^ patients with GBM and found that EBV seropositivity is associated with increased survival [46]. Thus, EBV infection and/or EBV-targeted immune responses may have some survival benefit for GBM patients. Another study found that 11 viral miRNAs are differentially expressed between glioma patients and healthy controls and that 6 of these are EBV miRNAs, suggesting that EBV infection may be associated with development of GBM [41].

On the tissue level, sometimes local EBV infection is found in patient gliomas. For instance, EBV was detected in 6/21 patient gliomas as determined by immunohistochemistry (IHC) and RNA in situ hybridization (ISH) in one study [86]. Direct sequencing revealed that 11/75 patient gliomas were EBV^+^ [87]. Another study demonstrated EBV positivity in 5/21 GBM samples [88]. In contrast, a next-generation sequencing study found no evidence of EBV genetic material in gliomas when analyzing 170 GBM datasets obtained from The Cancer Genome Atlas (TCGA) and an independent cohort of 61 GBM samples. Thus, the overall prevalence of EBV within gliomas varies widely between studies and at best is associated with a minority of GBM. The latent and lytic cycles of the virus and that 90% of the population is seropositive for EBV complicate claims about EBV status and associated glioma pathogenesis [2].

**Figure 3:**
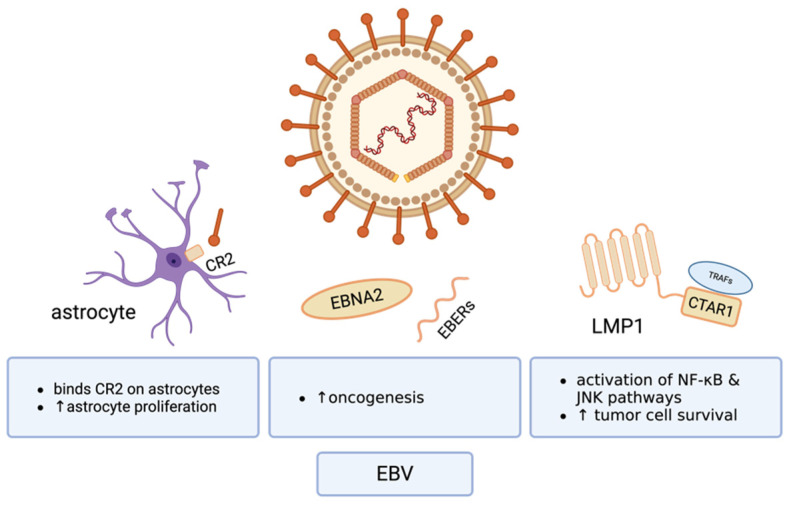
Studies of EBV seropositivity in GBM patients yield mixed reviews on patient prognoses. EBV may contribute to GBM progression by increasing astrocyte proliferation, by activating oncogenic pathways like NF-κB and JNK, and by increasing tumor cell survival. Created in BioRender. Hudson, AJ. (2025) https://BioRender.com/zhy5dfv (accessed on 12 June 2025).

On the cellular level, many theories have been proposed for how local EBV infection may affect glial cell morphology and oncogenesis (Figure 3). The major receptor for EBV, complement receptor 2 (CR2), is present on astrocytes, promotes entry of EBV into cultured astrocyte cell lines, and increases astrocytic proliferation [2]. Further EBV-derived viral products, i.e., EBV nuclear antigen 2 (EBNA2), EBV-encoded small RNAs (EBERs), and LMP1, contribute to oncogenesis in malignancies, including post-transplant lymphoproliferative disorder (PTLD), Hodgkin lymphoma (HL), diffuse large B cell lymphoma (DL-BCL), and nasopharyngeal carcinoma (NPC) [41,89]. Of special interest, EBV’s LMP1 binds TNF receptor-associated factor 6 (TRAF6), activating NF-κB and JNK and increasing cell survival [89]. When tumors were treated with pharmacological inhibitors of LMP1-activated NF-κB and Jun-N-terminal kinase (JNK), tumor cell death increased and tumor growth slowed [89]. Another EBV-targeted therapy that has successfully treated malignancies is the adoptive transfer of EBV-specific T cells, where EBV-targeted T cells are removed from patient blood, expanded ex vivo, and reintroduced [90]. In one clinical trial, the adoptive transfer of T cells targeted to EBV antigens EBNA1, MP1, and LMP2 sustained clinical responses in patients with local nasopharyngeal carcinoma [88]. These results, however, were limited in patients with metastatic disease [88]. Overall, the patient response rate was 48.7% [88]. Thus, given the likely presence of EBV in some gliomas and EBV’s known oncogenic properties in other malignancies, further study at the cellular level is needed to determine what mechanisms EBV may utilize to influence gliomagenesis and to discern if EBV-targeted therapies might benefit glioma patients.

### 4.4. CMV

Human cytomegalovirus (CMV/HHV-5) is a herpesvirus that encodes around 200 genes, in addition to microRNAs and non-coding transcripts [91,92]. CMV can infect and establish latency in many different cell types, including monocytes, macrophages, and myeloid lineage CD34^+^ hematopoietic progenitor cells [91,93]. CMV antibodies are present in about 60% of adults in developed countries and 100% of adults in the developing world [94]. While most CMV infections are asymptomatic, immunosuppression allows CMV in latently infected cells to reactivate, which can cause illness and worsen prognoses in immunosuppressed patients with GBM [94].

The effect of CMV infection on prognosis of GBM patients remains a topic of immense debate among scientists. Positive CMV serology and the presence of CMV intermediate early (IE) proteins on tumor pathology are associated with poor prognosis in GBM patients in some studies [95,96]. Other studies, however, show no significant impact of CMV infection on outcomes of GBM patients [46,97]. CMV’s presence in GBM tumors also remains controversial. In 2002, Dr. Charles Cobbs was among the first scientists to describe CMV gene products in human glioma samples [91,98]. Immunohistochemistry (IHC) and RNA in situ hybridization (ISH) showed CMV proteins and genetic material in 27 of 27 human glioma samples studied [98]. After Cobbs’s discovery, the field has been split on the prevalence of CMV in glioma [91,97]. Some studies find CMV DNA and protein are nearly ubiquitous in patient tumor samples [99,100,101]. However, other groups find no detectable CMV proteins or genetic materials in glioma [102,103]. One meta-analysis of 32 studies of more than 2000 patients determined that CMV detection in GBM varied dramatically depending on differences in detection methods and the populations studied [94,104]. Overall, this meta-analysis concluded that the CMV-positive rate of glioma patients is about 63% [94,104]. Another systematic review of 81 studies (7024 GBM samples, 2420 blood samples) found that CMV is expressed in 36% of GBM samples and 45.2% of blood samples of GBM patients [97,105]. To this day, though the exact extent of CMV expression in gliomas is disputed, CMV remains a virus of interest among scientists trying to better understand GBM.

**Figure 4:**
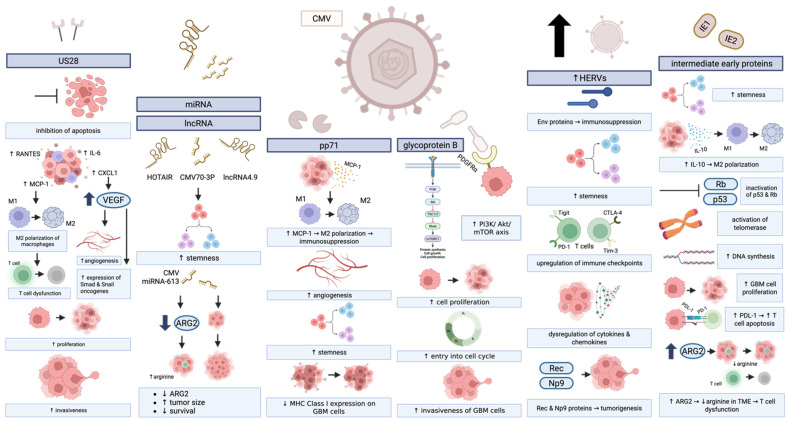
In GBM, CMV infection drives proliferation and invasiveness of GBM cells, increases stemness of GBM cells, contributes to T cell dysfunction, polarizes macrophages towards their suppressive M2 state, and dysregulates cytokines and chemokines, among its varied roles in gliomagenesis. Created in BioRender. Hudson, AJ. (2025) https://BioRender.com/l424xrm (accessed on 12 June 2025).

CMV infection of human GBM promotes tumorigenesis through various mechanisms (Figure 4). For instance, CMV infection increases activation of oncogenic pathways such as PI3K/Akt/mTOR, Wnt, NF-kB, EGFR, ERK, amphiregulin, and SOX-2, increasing cancer cell growth, survival, and metabolism [91,106]. CMV infection of GBM cells also activates telomerase, allowing infected cells to extend their telomeres and prolong survival [91,94,107]. In addition, CMV contributes to GBM cell stemness [92,94]. CMV-infected GBM cells display stemlike properties in culture, including slow growth, higher capacity for self-renewal, and increased resistance to TMZ compared to mock-infected controls [92]. Additionally, CMV-infected GSCs produce IL-10, resulting in the recruitment of immunosuppressive M2 macrophages to the TME [91,94,97]. CMV infection of GSCs also upregulates immunosuppressive PD-L1, enhances tumor stem cell migration, and promotes T cell apoptosis [94,108,109]. In addition, CMV-infected cells express viral intermediate early protein 1 (IE1), a viral protein that has many tumor-promoting effects within the tumor microenvironment [91].

Intermediate early proteins 1 and 2 (IE1 and IE2) are CMV-derived proteins that are co-expressed in GBM with stemness markers such as CD133, Nestin, and Sox2 [94,97]. IE1 expression in human GBM increases entry into the cell cycle, DNA synthesis, and proliferation [91]. Further, IE1 and IE2 inactivate p53 and Rb tumor suppressor genes, maintain S phase, and prevent apoptosis [97]. Knockout of IE1 and IE2 results in decreased Sox2 expression, decreased tumorsphere formation, and induction of apoptosis [97]. IE proteins can also activate telomerase, maintain mitotic cell cycle of host cells, and induce cell proliferation [94]. In addition, CMV-infected cells that express IE proteins have increased arginase 2 (ARG2) [91]. Overexpression of ARG2 by tumor cells can deplete arginine from the TME and contribute to T cell dysfunction as a result [110].

Expression of other CMV-derived proteins is also implicated in gliomagenesis. Glycoprotein B (gB) expression in glioma cells increases entry into the cell cycle and invasiveness of tumor cells [91]. gB binds receptor tyrosine kinase (RTK) PDGFR- α, which allows CMV to enter the cell. Viral cellular entry triggers the PI3k/Akt/mTOR axis, enhances cell proliferation, and drives gliomagenesis [97]. The CMV protein pp71 is preferentially expressed in CD133^+^ GSCs [91,97]. pp71 induces the pro-angiogenic stem cell factor (SCF) in GBM cells via NF-kB [97]. SCF then binds c-kit receptor tyrosine kinase, increasing endothelial progenitor cell migration and boosting angiogenesis [97]. Pp71 also transcriptionally upregulates the chemokine MCP-1 [97]. Additionally, pp71 decreases MHC class I expression on GBM cells, causing these cells to be overlooked by CD8^+^ T cells in the TME and interfering with anti-tumor immunity [91,97].

US28 is a virally encoded chemokine receptor that has many tumorigenic effects on GBM cells, and is present in 60% of human GBM samples [91]. US28 promotes GBM cell invasiveness and proliferation and blocks apoptosis [91]. US28 binds several chemokines, including MCP-1, RANTES, and CXCL1 [91]. CXCL1 overexpression enhances myeloid cell migration to the TME, disrupts T cell function, accelerates tumor progression, and confers poor prognosis to GBM patients [111]. Further, its overexpression leads to the secretion of VEGF, mediated by activation of hypoxia inducible factor 1 alpha/pyruvate kinase M2 (HIF-1alpha/PKM2) [91,97]. VEGF promotes angiogenesis and cancer stemness, among other roles [112]. Further, this increase in VEGF secretion induces glycogen synthase kinase 3 (GSK3) activity and activates transcription factors of oncogenes like Smads and Snail [94]. US28 expression is also associated with overexpression of IL-6 and activation of STAT3 [91,94,97]. Additionally, US28 upregulates endothelial nitric oxide synthase (e-NOS), which triggers upregulation of pro-invasive factors like ERK1, ERK2, FAK, and SRC and leads to increased GBM cell invasion [97]. By altering all the above pathways, US28 drives proliferation, invasion, angiogenesis, and metabolic reprogramming of GBM.

In addition, CMV infection induces transcription of human endogenous retroviruses (HERVs) [113]. HERVs are remnants of ancient viral infections that make up around 8% of the human genome [114]. They are generally transcriptionally active during embryogenesis and silenced during adulthood, but they can become activated in dysregulated states like cancer [113]. Retrovirus elements upregulated in CMV infection include HERV-T, HERV-W, HERV-F, and HERV-9 of the gammaretrovirus-related elements, HML2–4 and HML7–8 of the betaretrovirus elements, and the HERV-L group of Spuma-virus elements [113]. HERVs are thought to influence tumor development via immunosuppression by Env proteins and expression of regulatory proteins like Rec and Np9 that interact with cellular transcription factors involved in tumorigenesis [113,115]. Additionally, our lab showed that HERVs are upregulated in GSCs and are essential to their stemness, in part by inducing OCT4 [114]. The OCT4^+^ Sox2^+^ GSCs that HERV-K activation induces are immunosuppressive in nature. These cells upregulate immunosuppressive checkpoints PD-L1, CD70, A2aR, and TDO and dysregulate cytokines and chemokines [116]. Thus, another way in which CMV affects the GBM TME is by upregulating HERVS, contributing to immunosuppression, stemness, and treatment resistance in tumors.

CMV-associated miRNAs and long non-coding RNAs (lncRNA) also appear to play a role in tumorigenesis. For instance, CMV70-3P upregulates Sox2, inducing stemness in GBM cells [97]. CMV miRNA-613 decreases ARG2 and is associated with worse outcomes, including decreased survival and increased tumor size [91]. Additionally, CMV non-coding RNAs lncRNA4.9 and lncRNA HOTAIR directly interact with enzymatic subunit of zeste homolog 2 (EZH2), which is overexpressed in CMV-infected cells [93]. EZH2 is a downstream target of Myc and acts to expand the tumor stem cell pool in glioma, breast cancer, and prostate cancer and to promote proliferation, migration, and invasion in GBM [93].

Based on the substantial evidence that CMV enhances gliomagenesis, CMV has been the target of many recent therapeutics for GBM. Valganciclovir, an antiviral drug targeting CMV, inhibits viral DNA replication by competing with deoxyguanosine triphosphate to bind DNA polymerase [94]. In the Valcyte Treatment of Glioblastoma patients in Sweden (VIGAS) trial, the mean OS of patients treated for at least six months with valganciclovir doubled that of patients in a control group and that of patients treated for a shorter duration (24.1 months vs. 13.7 months and 13.1 months, respectively) [117]. The same research group followed up this study by analyzing outcomes of 28 different patients receiving valganciclovir as an add-on therapeutic. Patients receiving valganciclovir showed an almost doubled OS vs. control groups (25 months vs. 13.5 months). This study, however, was heavily criticized for bias in patient selection, limited characterization of the control group, and data analysis techniques [94,118]. Follow-up studies implementing valganciclovir as an add-on therapy also found OS prolongation by 5–7 months, though patients were not tested for CMV infection or viral load. These patients also differed in the treatments they received in addition to valganciclovir [94,119,120]. Therefore, although initial studies seem to show improved OS with valganciclovir, there remain some concerns surrounding the efficacy of this treatment.

Adoptive T cell therapy (ACT) is another widely studied CMV-targeting GBM treatment option [91]. In healthy patients, ~10% of CD8^+^ and CD4^+^ T are specific for CMV [97]. These CMV-specific T cells become dysfunctional in the GBM TME, characterized by decreased production of multiple cytokines, including IFN-γ, TNF, IL-2, and CD107a and increased exhaustion markers, such as PD-1, TIM-3, and CTLA-4 [97]. When co-cultured with autologous GBM cells, these CMV-specific CD8^+^ T cells elicit a potent anti-tumor effect [91,121]. Another therapeutic that has been developed and tested is vaccination with CMV gB and toll like receptor agonists [91]. Vaccination with gB leads to an influx of dendritic cells into draining lymph nodes, increased cross-presentation, and increase in adaptive immune response [91,122].

DC vaccination has been another form of targeting CMV in GBM patients. DCs are electroporated with pp65 mRNA and delivered to GBM patients, resulting in a powerful immune response when they migrate to draining lymph nodes [91]. A clinical trial combining pp65-electroporated DCs and tetanus diphtheria booster vaccination showed upregulation of CCL3, which promoted DC migration to vaccine site-draining lymph nodes, and increased overall survival of patients [91,123]. DC vaccines were also coupled with ACT in another clinical trial [91,124]. Patients who received combination treatment of pp65-specific T cells and DC vaccination showed increased CMV-specific CD8^+^ T cells, increased levels of IFN-γ, TNF-α, and CCL3, and increased survival compared to patients who received pp65-specific T cells and saline [91,124]. Combining pp65 DC vaccination with TMZ treatment and radiation also improved median progression-free survival and OS [91,125]. These findings support the notion that CMV is a worthy target for GBM research.

Thus, CMV alters the TME in GBM in various ways, including modifying cytokine levels, increasing expression of HERVs, causing immune dysregulation, promoting proliferation and invasiveness of GBM cells, and inducing stemness in GSCs. All these properties, while making GBM difficult to treat, may be outweighed by the ability of CMV to serve as a target of immunotherapeutics to transform the immunologically cold microenvironment of GBM into an immunologically hot one.

### 4.5. HHV-6

Human Herpesvirus 6 (HHV-6), also known as Roseolovirus and found in 90% of adults, has also been studied in connection with gliomas. HHV-6 was first discovered in the peripheral blood mononuclear cells (PBMCs) of patients with lymphoproliferative disorders or with AIDS in the 1980s [41]. HHV-6 has broad cellular tropism as it enters cells by binding CD46, which is present on all nucleated cells [41,42]. HHV-6 has been linked with several CNS diseases and hematological malignancies and has been studied in the context of gliomas [42]. As with other herpesviruses, the prevalence of HHV-6 in GBM tissue varies by study and by technique. In a 2001 study, HHV-6 DNA was detected in 14/31 gliomas, as well as in other normal and neoplastic nervous tissue [41,126]. Other studies report the presence of HHV-6 DNA in 1/21 gliomas, 17/40 gliomas (compared to 1/13 non-tumor autopsy controls), and 13/27 gliomas (compared to 5/30 controls) [41,88,127,128]. By protein detection, 47% of adult tumors were positive for viral protein U57, 24% of tumors expressed HHV-6 early antigen p41, and 35% of tumors contained HHV-6 late antigens, including gp116, gp64, and gp54 [41,129]. Thus, though there is variation in HHV-6 prevalence rates, all studies found evidence of HHV-6-positivity in at least some gliomas [88].

HHV-6 may contribute to gliomagenesis through various mechanisms. HHV-6 infects human oligodendrocytes and astrocytes both in vitro and in vivo, and HHV-6’s ORF-1 binds and inactivates p53 [42]. Further, ORF-1 transforms human epidermal keratinocytes and NIH 3T3 (mouse fibroblasts) cells in vitro [42]. In vivo, fibrosarcomas form after injection of ORF-1 into mice [42]. Additionally, HHV-6’s U95 protein binds and activates NF-κB, which further contributes to oncogenesis [42]. Also, the U24 protein, which is expressed in early stages of HHV-6 infection, is a cognate ligand for HNedd4L-WW3 (human neural precursor cell expressed developmentally downregulated protein 4-like) domain, whose dysregulation has been noted in gliomas [41]. Therefore, HHV-6 is found in human gliomas, and its viral products exhibit oncogenic function. For these reasons, further studies investigating its role in the glioma microenvironment are warranted.

## 5. Other Viruses

Several other viruses have been investigated for their potential role in gliomagenesis including the double-stranded DNA polyomaviruses John Cunningham virus (JCV), BK virus (BKV), and simian virus 40 (SV40). Polyomavirus infections are widespread—with over eighty percent of adults infected with JCV, BKV, or both—and are usually asymptomatic [130]. In immunocompromised individuals, JCV infection can cause multifocal leukoencephalopathy, a lethal demyelinating disease. Polyomaviruses have also been linked to various human malignancies, including colorectal cancer and Merkel cell carcinoma [1,130,131]. Notably, polyomavirus genetic material and proteins have been detected in glioma tissues. JCV was detected in 12/21, 7/13, and 11/21 patient GBM samples across several studies [132,133,134]. BKV was identified in 9/18 and 15/17 GBM samples in two separate studies [135,136]. SV40 infection was detected in 1/4, 4/8, and 6/16 GBM tumors using various techniques [137,138,139]. Although these findings suggest the presence of polyomavirus-derived products in some gliomas, their precise role in the GBM TME remains unclear.

These three viruses share an early viral gene product, T antigen, which has the ability to bind and inactivate tumor suppressors p53 and Rb and to transform neuronal cells in vitro [1,130,140]. Additionally, in animal studies, intracranial injection of these polyomaviruses induces brain tumors, including gliomas, medulloblastomas, and pineal gland tumors, at the injection site [1,140]. Nevertheless, despite evidence of polyomavirus gene products in gliomas and their tumorigenic potential in vitro and in animal models, a direct causal link between polyomaviruses and human brain tumors has not been established [1,130,140]. Their widespread prevalence in humans, along with the lack of a serological test for SV40, complicates efforts to clarify their roles in GBM, and population-level studies have found no significant links between infection with JCV or BKV and glioma risk [140,141].

Human papillomavirus (HPV), a well-established oncogenic virus, has also been detected in gliomas. One study detected HPV16 and HPV18 in 8/39 GBM samples, while another found HPV DNA in 12/52 GBM samples [142,143]. However, these studies did not explore HPV’s functional role in gliomas. Overall, although HPV viral gene products have been detected in some gliomas, mechanistic studies are needed to understand their potential role in the GBM TME.

## 6. Shared Mechanisms of Viral Oncomodulation

Individual viruses, such as CMV, HSV-1, EBV, HIV, and others exhibit distinct life cycles and cellular tropisms. However, there may be a degree of convergence on several related molecular and immunological pathways that may shape glioma oncogenesis and therapeutic resistance.

### 6.1. Immune Evasion and T Cell Exhaustion

Several of the viruses reviewed subvert host immune surveillance within the GBM microenvironment. HSV-1 latency-associated transcript (LAT) promotes CD8^+^ T cell exhaustion via PD-1 and TIM-3 upregulation [60]. CMV-infected glioma stem cells (GSCs) overexpress PD-L1 and secrete IL-10, suppressing anti-tumor immunity and recruiting immunosuppressive macrophages [91,94,97]. HIV induces an immunosuppressive state through CD4^+^ T cell depletion and local TGF-β2 upregulation, which inhibits MHC-II expression and T/NK cell function [7].

### 6.2. Activation of Oncogenic Pathways

Viral proteins from CMV (gB), HIV (p17, Tat), and EBV (LMP1) activate the PI3K/Akt/mTOR, NF-κB, and MAPK signaling pathways, all of which are critical in glioma pathogenesis and therapy resistance [35,84,91,94]. CMV IE1 also promotes entry into the cell cycle and sustains proliferation through EGFR and Wnt pathway activation [91,97,106].

### 6.3. Disruption of Tumor Suppressor Pathways

Multiple viral proteins inactivate key tumor suppressors such as p53 and Rb. CMV IE1/IE2 [97], HHV-6 ORF-1 [42], and HIV Nef [7,34] suppress p53-mediated apoptosis. Polyomavirus T antigens also directly bind and inhibit p53 and Rb [130,140].

### 6.4. Stemness and Plasticity

Stem-like properties are induced in infected glioma cells. CMV-infected GSCs express Sox2, CD133, and Nestin, and show increased resistance to temozolomide [92,94,97]. HIV-mediated oxidative stress enhances GSC frequency [24,30], while HHV-6 and HSV-1 may support stemness through NF-κB activation and oxidative stress pathways [42,52,91].

### 6.5. Epigenetic Reprogramming and Non-Coding RNAs

CMV miRNA-613 and lncRNAs such as HOTAIR interact with EZH2, a master regulator of glioma stemness [93]. HSV-1 miRNAs downregulate EPB41L1 and SH3PXD2A, activating Wnt/c-Myc signaling, and promoting migration and oxidative stress [64,66]. CMV also activates endogenous retroviruses (HERVs), such as HERV-K, which increase OCT4^+^ GSC populations and immune checkpoint expression [113,114,116].

These overlapping viral effects modulate oncogenesis and immunosuppression, independent of viral origin. Understanding these convergent mechanisms could inform common therapeutic targets across virus-associated gliomas.

## 7. Conclusions

This review highlights how a diverse set of viruses—including HIV, HSV-1, EBV, CMV, and others—may contribute to gliomagenesis not only by direct oncogenic transformation but also by remodeling the tumor immune landscape and supporting glioma stemness. These findings support a broader view of viral infections as active co-drivers of tumor biology in glioblastoma. Future research should focus on mapping virome signatures in GBM, therapies targeting shared viral mechanisms, investigating viral vectors in virotherapy, and leveraging viral immunogenicity. Ultimately, targeting virus-driven immune and oncogenic pathways holds promise for personalizing GBM treatment. With an integrated immunovirology approach, future therapies may not only suppress tumor progression but also stimulate durable anti-tumor immunity.

## Abbreviations

The following abbreviations are used in this manuscript:

GBM	Glioblastoma
OS	Overall survival
TME	Tumor microenvironment
Treg	Regulatory
MDSC	Myeloid-derived suppressor cell
HTLV-1	Human T-lymphocytic virus 1
ATL	Adult T cell leukemia
HAM/TSP	HTLV-1-associated myelopathy/ tropical spastic paraparesis
CNS	Central nervous system
GSC	Glioma stem cell
HIV	Human immunodeficiency virus
PLWH	People living with HIV
DC	Dendritic cell
NK	Natural killer
ART	Antiretroviral therapy
EBV	Epstein–Barr virus
IHC	Immunohistochemistry
TCGA	The Cancer Genome Atlas
PBMC	Peripheral blood mononuclear cells
HHV-6	Human Herpesvirus 6/ Roseolovirus
HSV-1	Herpes simplex virus 1
ROS	Reactive oxygen species
CMV	Cytomegalovirus
HERV	Human endogenous retrovirus
ACT	Adoptive T cell therapy
VZV	Varicella Zoster Virus
JCV	JC Virus
BKV	BK Virus
SV40	Simian Virus 40
HPV	Human Papillomavirus
